# Medullary Thyroid Cancer in Patients Older than 45—Epidemiologic Trends and Predictors of Survival

**DOI:** 10.3390/cancers12113124

**Published:** 2020-10-26

**Authors:** Shekhar Gogna, Michael Goldberg, David Samson, Mahir Gachabayov, Daniel M. Felsenreich, Asad Azim, Xiang D (Eric) Dong

**Affiliations:** 1 Section of Surgical Oncology, Department of Surgery, Westchester Medical Center/New York Medical College, Valhalla, NY 10595, USA; shekhar.gogna@wmchealth.org (S.G.); david.samson@wmchealth.org (D.S.); Mahir.Gachabayov@wmchealth.org (M.G.); moritz.felsenreich@meduniwien.ac.at (D.M.F.); asad.azim@wmchealth.org (A.A.); 2 Division of Endocrinology, Department of Medicine, Westchester Medical Center/New York Medical College, Valhalla, NY 10595, USA; michael.goldberg@wmchealth.org

**Keywords:** medullary thyroid carcinoma, thyroid cancer, thyroidectomy, survival, SEER, sporadic MTC

## Abstract

**Simple Summary:**

Sporadic medullary thyroid cancer can occur anytime in life although they tend to present at a later age (≥45 years old) when the tumors are more easily discernible or become symptomatic. We present, in this study, a group of patients diagnosed with medullary thyroid cancer at or after 45 years of age when they are more likely to develop sporadic forms of medullary thyroid cancer with regard to their natural history and prognosis. In this study, we evaluated factors affecting survival in such patients. We found that the incidence of medullary thyroid cancer in patients ≥45 years of age is increasing. Our findings suggest that patients should be offered surgical resection at an early stage to improve their outcomes.

**Abstract:**

Sporadic medullary thyroid cancer (MTC) can occur anytime in life although they tend to present at a later age (≥45 years old) when the tumors are more easily discernible or become symptomatic. We aimed to identify the factors affecting the survival in patients ≥45 years of age diagnosed with MTC. We analyzed the Surveillance, Epidemiology, and End Results (SEER) registry from 1973–2016 focusing on patients ≥45 years of age with MTC as an isolated primary. A total of 2533 patients aged ≥45 years with MTC were identified. There has been a statistically significant increase of 1.19% per year in the incidence of MTC for this group of patients. The disease was more common in females and the Caucasian population. Most patients had localized disease on presentation (47.6%). Increasing age and advanced stage of presentation were associated with worse survival with HR 1.05 (*p* < 0.001) and HR 3.68 (*p* < 0.001), respectively. Female sex and surgical resection were associated with improved survival with HR 0.74 (*p* < 0.001) and 0.36 (*p* < 0.001), respectively. In conclusion, the incidence of MTC in patients ≥45 years of age is increasing. Patients should be offered surgical resection at an early stage to improve their outcomes.

## 1. Introduction

Medullary thyroid carcinoma (MTC) is an uncommon malignancy accounting for approximately 5–10% of all thyroid cancers [1,2,3]. More specifically, sporadic MTC accounts for approximately 75% of all MTC, diagnosed in the United States yearly, while hereditary MTC is responsible for the remaining 25% of the cases [4,5,6,7]. The age of onset for sporadic MTC is much more variable than hereditary MTC, albeit often at a later age in patients [4,8,9]. Management of patients with MTC frequently involves first identifying patients with undetected germline RET mutations along with determination of the extent of disease in newly diagnosed MTC patients. As a group, their prognosis is worse than differentiated thyroid cancer (DTC) and frequently involves patients developing nodal and occasional systemic disease following diagnosis.

Somatic mutations or gene rearrangements in the RET proto-oncogene occurs in approximately 40–75% of the cases diagnosed in the sporadic MTC group [3,10]. The overall prevalence of somatic RET mutation is somewhat lower than expected, but codon 634 still comprises a majority of the RET mutation similar to MEN2A patients [11,12]. Patients with sporadic MTC tend to have larger tumors, advanced stage, nodal metastases, and overall worse outcome [3,13,14]. Alternatively, the development of MTC can occur through a RAS mutation, which is generally exclusive for sporadic, non-hereditary, MTC. The most common type of mutation not related to RET mutation is the HRASQ61R HRAS mutation occurring in up to 11% of sporadic MTC cases, which is thought to be associated with an intermediate level of aggressiveness in terms of tumor progression [15,16].

Historically, only DTC prominently features age as a discriminating factor in terms of prognosis. More recently, age is becoming a more notable prognostic indicator in patients with sporadic MTC [4,8,9]. Through the use of the SEER database from 2000 till 2010, age has been identified as a key component of a nomogram to prognosticate MTC patients [8]. Using data from the Surveillance, Epidemiology, and End Results (SEER) database, Qu et al. were able to generate two nomograms, one based on age cutoff of 49 and 69 years of age, and another with 45 years of age as the cutoff. Therefore, although not part of the current American Joint Committee on Cancer (AJCC) staging, age is increasingly being recognized as an important prognostic indicator [8].

Patients with sporadic MTC have not been well studied as a group due to the difficulty in isolating them as a group. Sporadic MTC presents a unique challenge to the treating physician. The treating physician needs to exclude familial syndromes first, which may give rise to MTC. Familial cases of MTC need to be identified in order to exclude undiagnosed associated endocrinopathies. This will also allow family members to be treated early on. Subsequently, patients with isolated sporadic MTC still present a management challenge due to the unfavorable prognosis compared with DTC [17,18].

The older sporadic MTC patient is sometimes overlooked although they comprise the larger proportion of the patients with MTC. To isolate the patients in the SEER database presenting with sporadic MTC, we chose to use the age cutoff of 45 to capture the majority of patients with sporadic MTC. The SEER database has one of the largest cohort of patients with MTC. We explored this specific group of patients (≥45 years of age) in an attempt to better understand the natural history of patients with sporadic MTC.

## 2. Results

### 2.1. Epidemiology and Cancer-Free Survival Rate

**Figure 1:**
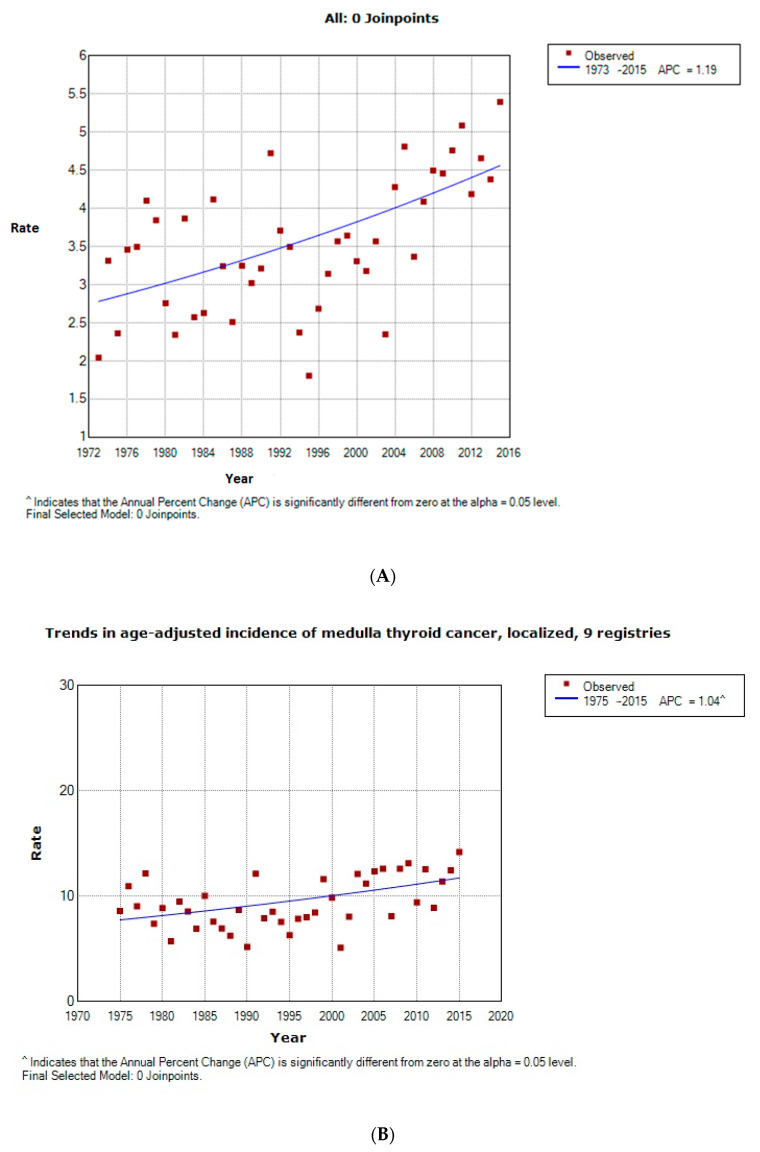

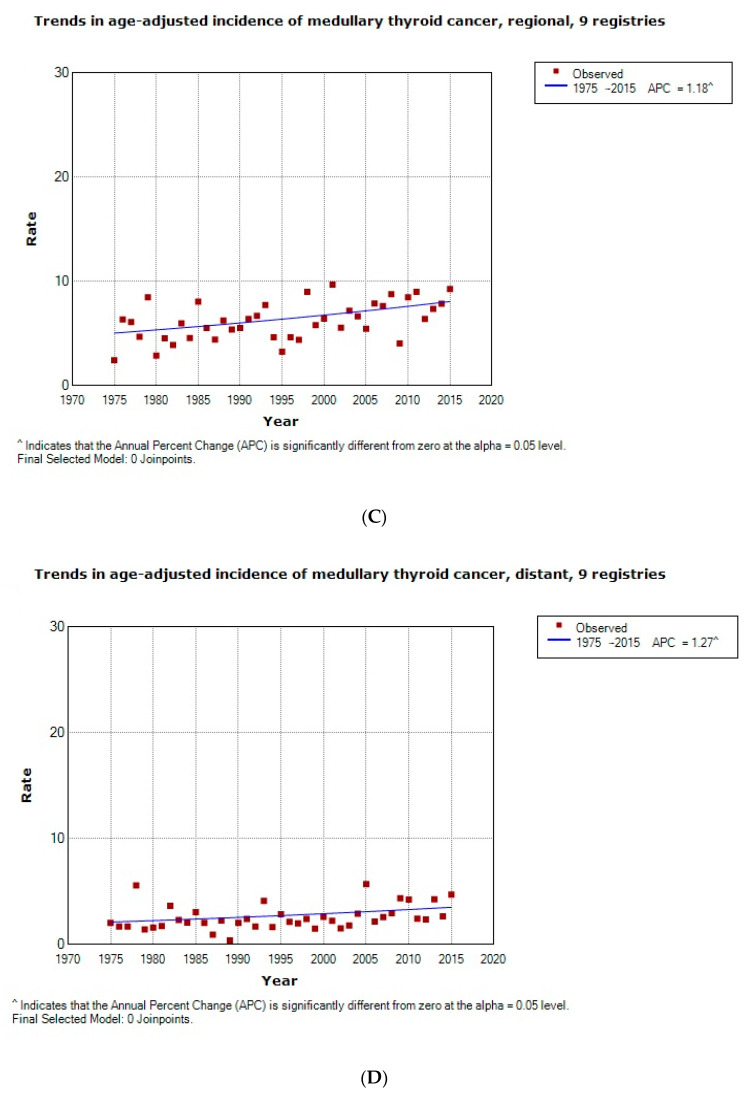
(**A**) Overall trend. (**B**) Trend observed in the localized stage. (**C**) Trend observed in the regional stage. (**D**) Trend observed in the distant stage.

Demographics of the included patients are summarized in Table 1. A total of 2533 patients with MTC were identified from the SEER database between the years 1973–2016, with a mean age of 60.1 ± 10.7 years. There has been a statistically significant increase in age-adjusted localized MTC by 1.19% per year including all stages from the years 1973–2015 (Figure 1A). When accounting for the extent of the disease, localized disease in this group of the patient has increased by 1.04% (Figure 1B), regional MTC increased by 1.18% per year (Figure 1C), and the incidence of MTC with distant metastasis increased by 1.27% per year (Figure 1D) over the past 43 years. 

### 2.2. Survival Based on Demographics

**Figure 2:**
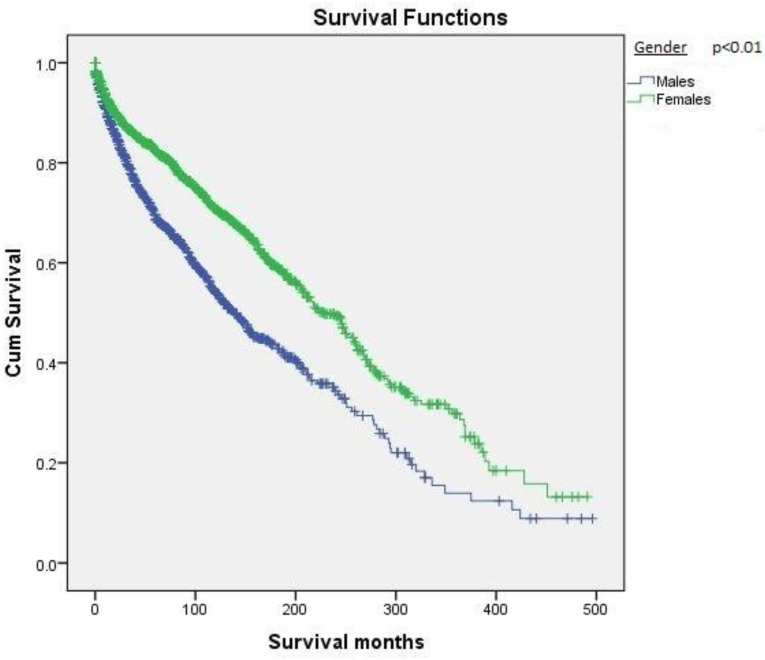
Kaplan–Meier survival curves based on gender.

**Figure 3:**
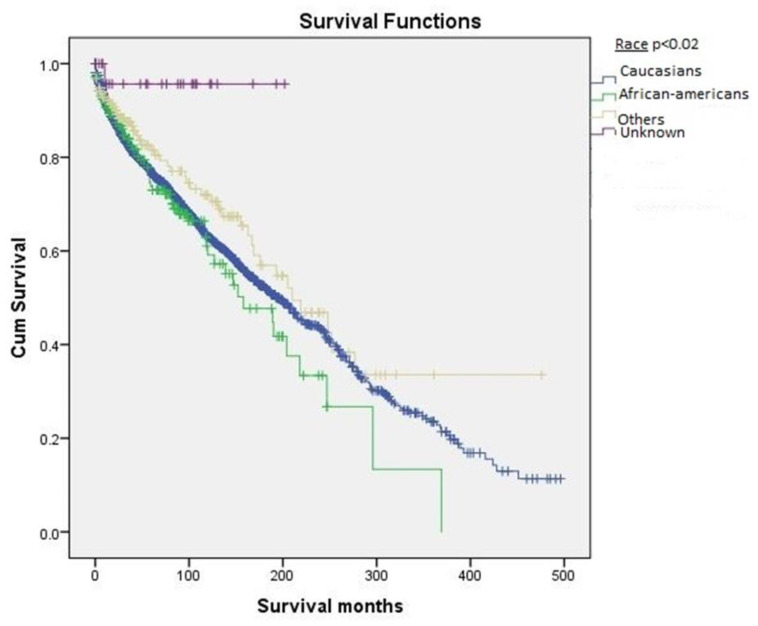
Kaplan–Meier survival curves based on race.

Table 1 shows the demographic distribution and survival based on the reported variables. Females outnumbered males in both number (58.6% vs. 41.4%) and overall survival (239.0 ± 7.5 vs. 180.7 ± 8.4 months) (Figure 2). In terms of racial distribution, Caucasians represented the largest cohort (*n* = 2172, 85.7%) followed by African-Americans (*n* = 177, 7.0%), and other minority races (Native Americans, Asian/Pacific islanders, *n* = 156, 6.2%). The survival based on racial distribution was highest in Asians/Native Americans, followed by Caucasians (Figure 3). When the patients were divided into different age groups, younger patients had better survival than older patients.

### 2.3. Tumor Description and Survival Characteristics

The mean tumor size was 25.6 ± 2.7 mm (range 1–99.5 mm) (Table 2). A mean of 20.0 ± 20.2 cervical lymph nodes were isolated during surgery and approximately 5.75 ± 11.07 nodes were positive for metastasis, indicating the majority of lymph nodes were positive following diagnosis based on surgical pathology. 

**Figure 4:**
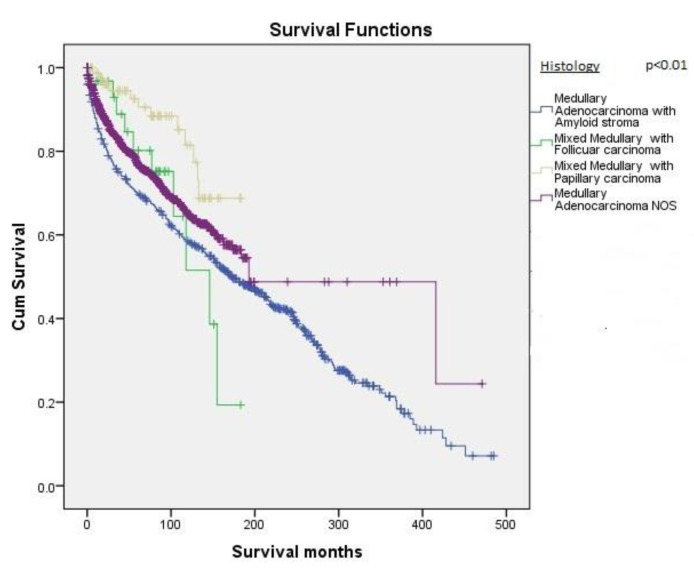
Kaplan–Meier survival curves based on the type of tumor histology.

In the histopathological subgroup analysis, MTC NOS (*n* = 1704, 67.3%) was the most common variant with the best survival followed by MTC with an amyloid stoma (*n* = 702, 27.7%). MTC with the papillary variant (*n* = 89, 3.5%) and MTC with follicular variant (*n* = 38, 1.5%) were less common and also showed worse survival, respectively. This difference in survival based on histopathological subtype was statistically significant (*p* < 0.01) (Figure 4).

The extension of the tumor also impacted survival. The most common type of tumor extension was a single invasive tumor confined to the thyroid associated with the best survival (*n* = 955, 37.7%). This was followed by in situ invasion (*n* = 464, 18.3%), extension into the thyroid capsule (*n* = 317, 12.5%), and multiple foci confined to the thyroid gland (*n* = 275, 10.9%), respectively. Other less common patterns of tumor extension are also listed in Table 3 and included direct extension into surrounding structures. Together, they account for about 7.5% (*n* = 190) of patients. The survival gradually worsened with advanced tumor extension (*p* < 0.01).

**Figure 5:**
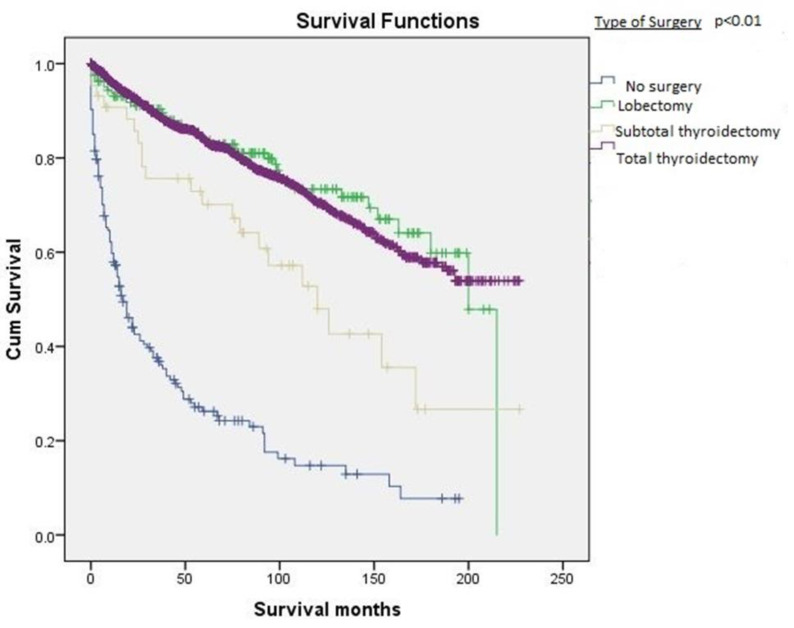
Kaplan–Meier survival curve based on the type of surgical resection.

Patients who underwent total thyroidectomy had the best survival (165.3 ± 2.7 months), followed by patients who had lobectomy with or without ipsilateral thyroidectomy (162.1 ± 7.1 months), than those who had a subtotal thyroidectomy (122.2 ± 14.4 months). Patients who did not receive surgery had the poorest survival (47.3 ± 5.3 months) (Figure 5).

### 2.4. Cox Proportional Hazard Model

The results of the stratified Cox model are shown in Table 3. Increasing age, male gender, number of positive cervical lymph nodes, patients who did not receive surgical resection, and distant metastasis were all associated with a higher risk of mortality.

## 3. Discussion

The incidence of sporadic MTC among the population greater than or equal to 45 years of age is rising nationally. This group of patients frequently present with advanced stage disease, higher tumor burden, and more nodal metastasis. Based on an analysis of this group of patients, early diagnosis, along with surgical intervention, is associated with improved survival.

Medullary thyroid carcinoma was first described as a distinct histologic entity in 1959 [19]. This early report of MTC found a high incidence of lymph node metastases (12/21 cases) along with an intermediate grade of malignancy when compared with DTC and anaplastic thyroid cancers. Out of the 21 cases described, the median age was 50 years, two-thirds were women, and eight out of twenty-one patients developed distant metastases following initial diagnosis [19]. Furthermore, they were able to determine that the presence of amyloid was paramount for the characterization of this tumor type [19]. Additionally, as the tumor required a separate terminology from papillary or follicular and anaplastic tissue types, the word medullary, used in other fields such as botany, was chosen based on the similar histologic pattern and having a better description than the term “solid” carcinoma can convey [19].

Subsequent investigations into the pathophysiology of MTC revealed its relationship with familial MTC and MEN syndromes [20,21,22,23,24,25,26,27]. Germline mutations in the RET proto-oncogene was studied extensively and led to early treatment recommendations for patients harboring the mutation [21,22,23,24,25,26,27]. Current American Thyroid Association (ATA) guidelines for MTC are based on genetic karyotyping with its well-known phenotypic presentations and associated endocrinopathies [11]. For sporadic MTC, the diagnosis can be more challenging. Due to the presence of calcitonin elaboration, patients being evaluated for thyroid nodules are sometimes considered for calcitonin level measurements, based on several European association guidelines [11,20]. Although popular in Europe, variations in the European guidelines and ATA guidelines as a screening modality for thyroid nodules in older patients never led to its widespread adoption in the U.S. [11,28].

The typical age of presentation for sporadic MTC is in the fifth decade of life [18,29,30,31]. In contrast, patients with MEN 2A and FMTC typically present with MTC in the third decade of life while MEN 2B develops MTC in those younger than 20 years of age [18,29,30,31]. As the incidence of MTC is nearly 100% in familial cases, patients who develop MTC after 45 years of age are likely to be of the sporadic type. Several studies have looked into the characteristics of this group of patients in particular. Based on the Irish National Cancer Registry, Lennon et al. found this group of patients to have a median age of 52 from a group of 43 patients. [9] Notably, this group of patients had a 10-year survival of 48.63%, which is worse than the traditional cohort of patients seen with MTC found with MEN syndromes. Only two of the 43 patients had RET proto-oncogene defects associated with their disease. Due to the lack of widespread screening for thyroid cancer, 65.1% of the patients in the Irish National Cancer Registry had advanced stage III or IV disease on presentation [9]. Similarly, Jayakody et al. looked into their database from Sydney and found a higher incidence of advanced-stage disease in sporadic MTC compared with familial MTC [32]. The patients with sporadic MTC also presented with a median age of 55.4 compared with 43.9 in patients with MEN2 syndrome [32]. Patients also tended to have larger size tumors and a higher incidence of lymph node metastases. Their preoperative calcitonin is higher and survival is worse, perhaps due to the delay in diagnosis seen in this cohort of patients [32].

In order to study patients with MTC without germline RET mutation without resorting to germline testing, we elected to study the patient population greater than or equal to 45 years of age [29]. Compared to other published studies, the group of patients found in the SEER database showed that they were slightly older than previously recognized. The mean age of presentation was 60.1 years old, although one has to bear in mind that younger patients were excluded from the analysis, assuming that most of them had associated MEN syndrome or FMTC. The patients were mostly Caucasian, although the disease is also present in other races with the prognosis somewhat worse in African-Americans. A majority of patients do present with localized disease and are offered surgery, which improves their outcome.

Patients with MTC in this study had a high incidence of disease confined to the thyroid. However, despite 85.9% of patients having the disease confined to the thyroid, a high number showed positive lymph nodes for metastatic thyroid cancer. A large percentage of patients (78.9%) underwent total or subtotal thyroidectomy as primary treatment for this disease.

Our study has a few advantages. It is the biggest study to date on sporadic MTC. We used the SEER database and the information was gathered from multiple SEER databases and registries, which allows for the inclusion of a large sample population. However, it does add to the heterogeneity of the dataset. We used a unique epidemiological tool in the form of a Joinpoint regression-based method to assess the age-adjusted trend of this pathology across various stages in patients. There are few inherent limitations to the study. First, it is retrospective in nature. The clinical follow-up, adjuvant treatment in the form of chemotherapy and radiation, post-operative morbidity/mortality, and radiological imaging are not available in this dataset. The SEER database is unable to provide information on the genetic constitution of the patient population. Finally, data on disease progression is limited, which prevents the formulation of strong recommendations on follow-up, surveillance, and treatment.

## 4. Materials and Methods

This is a retrospective cohort study from the SEER database using 18 registries to identify all patients aged ≥ 45 years from 1973–2016, using SEER site-specific primary code, based on the International Classification of Diseases for Oncology, Third Edition (ICD-O-3). Medullary carcinoma of the thyroid was identified by using the ICD-O-3 code for the primary site (C73.9). The histology codes used for MTC histopathology were extracted from ICD-O-3 (Supplementary Table S1).

### 4.1. Inclusion Criteria

All patients with isolated MTC ≥ 45 years of age were included in the study.

### 4.2. Exclusion Criteria

Patients that were <45 years old were excluded. Patients with any histopathology other than MTC were excluded. Patients with more than one concurrent malignancy were excluded. Patients with incomplete or missing data were excluded.

### 4.3. Patient-Related Variables

The data were collected for age, gender, and race. Tumor related variables such as histopathological subclasses, grade, size and extension of tumor, SEER historic stage (the SEER program tries to make all localized/regional/distant stage variables consistent for all cancer sites for the appropriate years), number of involved lymph nodes, and type of surgical resection were collected. Survival time after diagnosis in months, and the status of the patient in terms of being alive or deceased was also noted.

### 4.4. Outcome Measures

The primary objective was to identify the predictors of mortality. The secondary objective was to analyze differences in survival based on age, gender, race, tumor characteristics, tumor grade, histopathological subclass, tumor extent, lymph node status, SEER based stage, and types of surgical resection. Changes in the epidemiological trend in the U.S. over the last four decades were also analyzed.

We used the frequency and survival session from SEERStat version 8.3.5 to observe incidence and trend analysis over four-decades in the U.S. in this age group. Joinpoint software 6.0.0 was utilized to analyze the age-adjusted epidemiological trends at the national level. The software takes trend data (e.g., cancer rates) and fits the simplest Joinpoint model that the data allow. This enables the user to test whether an apparent change in trend is statistically significant [33]. The data on epidemiological trend was calculated using Joinpoint from the years 1973–2015. Patients with missing data were excluded from the analysis.

### 4.5. Survival Analysis

In the first step of data analysis, due to the survival nature of the outcome data, preliminary analyses such as the Kaplan–Meier log-rank tests were performed. Subsequently, we utilized the Cox proportional hazard model for predicting and refining patient survival rates. The overall survival (OS) rates were calculated using the actuarial (Kaplan–Meier) method. Differences in survival based on age, sex, histology, tumor extent, lymph node status, grade, SEER stage, and surgery was computed using a log-rank test (Mantzel–Cox). Multivariable analysis was conducted using Cox regression for proportional hazards by backward elimination to identify the independent effect of covariates on survival controlling for the previously mentioned variables.

All *p*-values were 2-sided and a *p*-value < 0.05 was considered significant. Statistical analysis was conducted using SPSS for IBM Corp, released 2017 (IBM SPSS Statistics for Windows, Version 26.0. IBM Corp; Armonk, NY, USA).

According to the hospital policy, this study was approved by the Institutional Review Board of New York Medical College (IRB Log Number: 12,996). Patient consent form is not applicable as the SEER database was analyzed retrospectively. This retrospective observational study is reported according to Strengthening The Reporting of Observational studies in Epidemiology (STROBE) guidelines [34].

## 5. Conclusions

The incidence of MTC in patients aged ≥ 45 years is increasing. Patients undergoing surgical resection at the early stage have improved outcomes. Advanced age and higher tumor burden are detrimental to overall survival.

## Figures and Tables

**Table 1:** Demographics and survival of the patient population.

Variables	N (%)	Survival (Months)	*p*-Value
Age			
45–64 years	1362 (53.7%)	254.2 ± 7.3	
65–84 years	772 (30.4%)	124.5 ± 6.5	<0.01
≥85 years	399 (15.7%)	43.6 ± 6.5	
Gender			<0.01
Males (N, %)	1049 (41.4%)	180.7 ± 8.4	
Females (N, %)	1484 (58.6%)	239.0 ± 7.5	
Race			0.02
Caucasians	2172 (85.7%)	215.1 ± 6.0	
African-Americans	177 (7.0%)	173.0 ± 16.0	
Others (American Indians, Asian Pacific islanders)	156 (6.2%)	251 ± 16.3	
Unknown	28 (1.1%)	194 ± 8.1	

**Table 2:** Description of tumor characteristics and survival.

Variables	Mean	SD	*p*-Value
Tumor size (±SD)	25.6 (mm)	2.7 (mm)	
Number of cervical lymph nodes isolated (N ± SD)	42.4	97.6	
Number of cervical lymph nodes positive for metastasis (N ± SD)	36.0	44.6	
	N (%)	Survival (months)	
Histological subtypes			<0.01
Medullary adenocarcinoma NOS	1704 (67.3%)	250 ± 16	
Medullary adenocarcinoma with an amyloid stoma	702 (27.7%)	202.6 ± 7.0	
Medullary adenocarcinoma with papillary variant	89 (3.5%)	156.5 ± 6.7	
Medullary adenocarcinoma with follicular variant	38 (1.5%)	119.3 ± 12.4	
Tumor extension			<0.01
In-situ: Noninvasive	464 (18.3%)	172.6 ± 9.4	
Single invasive tumor confined to the thyroid	955 (37.7%)	243 ± 6.2	
Multiple foci confined to the thyroid	275 (10.9%)	229.6 ± 12.4	
Localized, NOS	165 (6.5%)	171.2 ± 11.2	
Into thyroid capsule, but not beyond	317 (12.5%)	179 ± 11.2	
Pericapsular soft/connective tissue, Parathyroid Strap muscle(s), Nerves: Recurrent laryngeal, vagus	97 (3.8%)	136.1 ± 12	
Extension to: Major blood vessel(s), SCM muscle Esophagus, Larynx (The tumor is described as fixed to adjacent tissues)	23 (0.9%)	80.1 ± 13	
Trachea, Skeletal muscle, other than strap or SCM muscle, Bone	29 (1.1%)	46.3 ± 9.1	
Extension into Mediastinal tissues	41 (1.6%)	55.3 ± 9.0	
Metastasis	76 (3.0%)	37.0 ± 7.0	
Unknown if extension present	91 (3.4%)	62.0 ± 7.7	
Type of surgery performed			<0.01
Total thyroidectomy	1655 (65.3%)	165.3 ± 2.7	
Subtotal thyroidectomy	344 (13%)	122.2 ± 14.4	
Thyroid Lobectomy	262 (10%)	162.1 ± 7.1	
No surgery	272 (10%)	47.3 ± 5.3	

**Table 3:** Cox regression model predicting mortality for patients (age ≥ 45 years) with medullary carcinoma thyroid.

Factors	Hazard Ratio	95% CI	*p*-Value
Age	1.058	1.049–1.069	<0.01
Gender			
Females (Reference)	1		
Males	1.204	1.009–1.437	0.04
Nodal Status			
Negative cervical nodes			
Positive cervical lymph nodes	1.003	1.001–1.006	<0.01
Type of Surgical resection			
Total thyroidectomy (Reference)			
Subtotal thyroidectomy	0.289	0.166–0.317	<0.01
Thyroid Lobectomy	0.832	0.392–1.117	0.30
No surgery	2.180	1.390–3.419	<0.01

## Supplementary Materials

The following are available online at https://www.mdpi.com/2072-6694/12/11/3124/s1, Table S1: Histological subtypes of MTC.

## References

[1] Ho A.S., Wang L., Palmer F.L., Yu C., Toset A., Patel S., Kattan M.W., Tuttle R.M., Ganly I. (2015). Postoperative Nomogram for Predicting Cancer-Specific Mortality in Medullary Thyroid Cancer. Ann. Surg. Oncol..

[2] Miyauchi A., Matsuzaka F., Hirai K., Yokozawa T., Kobayashi K., Ito Y., Nakano K., Kuma K., Futami H., Yamaguchi K. (2002). Prospective trial of unilateral surgery for nonhereditary medullary thyroid carcinoma in patient without germline RET mutations. World J. Surg..

[3] Larouche V., Akirov A., Thomas C.M., Krzyzanowska M.K., Ezzat S. (2019). A primer on the genetics of medullary thyroid cancer. Curr. Oncol..

[4] Randle R.W., Balentine C.J., Leverson G.E., Havlena J.A., Sippel R.S., Schneider D.F., Pitt S.C. (2017). Trends in the presentation, treatment, and survival of patients with medullary thyroid cancer over the past 30 years. Surgery.

[5] Viola D., Elisei R. (2019). Management of medullary thyroid cancer. Endocrinol. Metab. Clin..

[6] Moo-Young T.A., Traugott A.L., Moley J.F. (2009). Sporadic and familial medullary thyroid carcinoma, state of the art. Surg. Clin..

[7] Fallah M., Sundquist K., Hemminki K. (2013). Risk of thyroid cancer in relatives of patients with medullary thyroid carcinoma by age at diagnosis. Endocr.-Relat. Cancer.

[8] Qu N., Shi R.L., Luo T.X., Wang Y.L., Li D.S., Wang Y., Huang C.P., Ji Q.H. (2016). Prognostic significance and optimal cutoff of age in medullary thyroid cancer. Oncotarget.

[9] Lennon P., Deady S., White N., Lambert D., Healy M.L., Green A., Kinsella J., Timon C., O’Neill J.P.O. (2017). Aggressive medullary thyroid cancer, an analysis of the Irish National Cancer Registry. Ir. J. Med. Sci..

[10] Gundara J.S., Zhao J., Gill A.J., Lee J.C., Delbridge L., Robinson B.G., McLean C., Serpell J., Sidhu S.B. (2015). Noncoding RNA blockade of autophagy is therapeutic in medullary thyroid cancer. Cancer Med..

[11] Patel K.N., Yip L., Lubitz C.C., Grubbs E.G., Miller B.S., Shen W., Angelos P., Chen H., Doherty G.M., Fahey T.J. (2020). Executive summary of the American Association of Endocrine Surgeons Guidelines for the Definitive surgical management of thyroid disease in adults. Ann. Surg..

[12] Wells S.A., Asa S.L., Dralle H., Elisei R., Evans D.B., Gagel R.F. (2015). Revised American Thyroid Association Guidelines for the Management of Medullary Thyroid Carcinoma. Thyroid.

[13] Rich T.A., Feng L., Busaidy N., Cote G.J., Gagel R.F., Hu M., Jimenez C., Lee J.E., Perrier N., Sherman S.I. (2014). Prevalence by Age and Predictors of Medullary Thyroid Cancer in Patients with Lower Risk Germline RET Proto-Oncogene Mutations. Thyroid.

[14] Figlioli G., Landi S., Romei C., Elisei R., Gemignani F. (2013). Medullary thyroid carcinoma (MTC) and RET proto-oncogene: Mutation spectrum in the familial cases and a meta-analysis of studies on the sporadic form. Mutat. Res..

[15] Moura M.M., Cavaco B.M., Leite V. (2015). RAS proto-oncogene in medullary thyroid carcinoma. Endocr. Relat. Cancer.

[16] Yamada T., Iwai T., Takahashi G., Kan H., Koizumi M., Matsuda A., Shinji S., Yamagishi A., Yokoyama Y., Tatsuguchi A. (2016). Utility of KRAS mutation detection using circulating cell-free DNA from patients with colorectal cancer. Cancer Sci..

[17] Fink M., Weinhausel A., Niederle B., Hass O.A. (1996). Distinction between sporadic and hereditary medullary thyroid carcinoma (MTC) by mutation analysis of the RET proto-oncogene. Int. J. Cancer.

[18] Roy M., Chen H., Sippel R.S. (2013). Current understanding and management of medullary thyroid cancer. Oncologist.

[19] Hazard J.B., HAWK W.A., CRILE J.R.G. (1959). Medullary (solid) carcinoma of the thyroid; a clinicopathologic entity. J. Clin. Endocrinol. Metab..

[20] Machens A., Dralle H. (2016). Surgical cure rates of sporadic medullary thyroid cancer in the era of calcitonin screening. Eur. J. Endocrinol..

[21] Grubbs E.G., Williams M.D., Scheet P., Vattathil S., Perrier N.D., Lee J.E., Gagel R.F., Hai T., Feng L., Cabanillas M.E. (2016). Role of CDKN2C copy number in sporadic medullary thyroid carcinoma. Thyroid.

[22] Takahasi M., Ritz J., Cooper G.M. (1985). Activation of a novel human transforming gene, ret, by DNA rearrangement. Cell.

[23] Pasini A., Geneste O., Legrand P., Schlumberger M., Rossel M., Fournier L., Rudkin B.B., Schuffenecker I., Lenoir G.M., Billaud M. (1997). Oncogenic activation of RET by two distinct FMTC mutations affecting the tyrosine kinase domain. Oncogene.

[24] Mulligan L.M., Kwok J.B., Healey C.S., Elsdon M.J., Eng C., Gardner E., Love D.R., Mole S.E., Moore K.K., Papi L. (1993). Germline mutations of the RET proto-oncogene in multiple endocrine neoplasia type 2A. Nature.

[25] Donis-Keller H., Dou S., Chi D., Carlson K.M., Toshima K., Lairmore T.C., Howe J.R., Moley J.F., Goodfellow P., Wells S.A. (1993). Mutations of the RET proto-oncogene are associated with MEN 2A and FMTC. Hum. Mol. Genet..

[26] Elisei R., Cosci B., Romei C., Bottici V., Renzini G., Molinaro E., Agate L., Vivaldi A., Faviana P., Basolo F. (2008). Prognostic significance of somatic RET oncogene mutations in sporadic medullary thyroid cancer: A 10-year follow-up study. J. Clin. Endocrinol. Metab..

[27] Accardo G., Conzo G., Esposito D., Gambardella C., Mazzella M., Castaldo F., Donna C.D., Polistena A., Avenia N., Colantuoni V. (2017). Genetics of medullary thyroid cancer: An overview. Int. J. Surg..

[28] Pacini F., Schlumberger M., Dralle H., Elisei R., Smit J.W.A., Wiersinga W. (2006). European consensus for the management of patients with differentiated thyroid carcinoma of the follicular epithelium. Eur. J. Endocrinol..

[29] Farndon J.R., Leightt G.S., Dilley W.G., Baylin S.B., Smallridge R.C., Harrison T.S., Wells S.A. (1986). Familial medullary thyroid carcinoma without associated endocrinopathies: A distinct clinical entity. Br. J. Surg..

[30] Kebebew E., Ituarte P.G., Siperstein A.E., Duh Q.Y., Clark O.H. (2000). Medullary Thyroid Carcinoma: Clinical Characteristics, Treatment, Prognostic Factors, and a Comparison of Staging Systems. Cancer.

[31] Roman S., Lin R., Sosa J.A. (2006). Prognosis of Medullary Thyroid Carcinoma: Demographic, Clinical, and Pathologic Predictors of Survival in 1252 Cases. Cancer.

[32] Jayakody S., Reagh J., Bullock M., Aniss A., Clifton-Bligh R., Learoyd D., Robinson B., Delbridge L., Sidhu S., Gill A.J. (2018). Medullary thyroid carcinoma: Survival analysis and evaluation of mutation-specific immunohistochemistry in the detection of sporadic disease. World J. Surg..

[33] Kim H.J., Fay M.P., Feuer E.J., Midthune D.N. (2000). Permutation tests for joinpoint regression with applications to cancer rates. Stat. Med..

[34] Von Elm E., Altman D.G., Egger M., Pocock S.J., Gøtzsche P.C., Vandenbroucke J.P. (2007). The strengthening the reporting of observational studies in epidemiology (STROBE) statement: Guidelines for reporting observational studies. Ann. Intern. Med..

