# Supplementary material for: Medullary Thyroid Cancer in Patients Older than 45—Epidemiologic Trends and Predictors of Survival
Source: Cancers (Basel). 2020 Oct 26;12(11):3124. doi: 10.3390/cancers12113124 (PMC7692716; doi:10.3390/cancers12113124)
Supplement: Supplementary file 1 [file cancers-12-03124-s001.pdf]

cancers

Supplementary Table

Medullary Thyroid Cancer in Patients Older than 45—Epidemiologic Trends and Predictors of Survival

Shekhar Gogna, Michael Goldberg, David Samson, Mahir Gachabayov, Daniel M. Felsenreich, Asad Azim and Xiang D (Eric) Dong

Table 1. Histological subtypes of MTC.

Histological subtypes of MTC	ICD – O - 3 Histology code
Medullary carcinoma with amyloid stroma	8345
Medullary carcinoma with follicular variant	8346
Medullary carcinoma with papillary variant	8347
Medullary carcinoma NOS	8510
Medullary carcinoma with lymphoid invasion	8512
